# Development of 3D-Printed Sulfated Chitosan Modified Bioresorbable Stents for Coronary Artery Disease

**DOI:** 10.3389/fbioe.2020.00462

**Published:** 2020-05-19

**Authors:** Tianyang Qiu, Wei Jiang, Pei Yan, Li Jiao, Xibin Wang

**Affiliations:** ^1^Key Laboratory of Fundamental Science for Advanced Machining, Beijing Institute of Technology, Beijing, China; ^2^School of Mechanical Engineering, Beijing Institute of Technology, Beijing, China

**Keywords:** bioresorbable stent, 3D printing, sulfated chitosan, mechanical property, biocompatibility

## Abstract

Bioresorbable polymeric stents have attracted great interest for coronary artery disease because they can provide mechanical support first and then disappear within a desired time period. The conventional manufacturing process is laser cutting, and generally they are fabricated from tubular prototypes produced by injection molding or melt extrusion. The aim of this study is to fabricate and characterize a novel bioresorbable polymeric stent for treatment of coronary artery disease. Polycaprolactone (PCL) is investigated as suitable material for biomedical stents. A rotary 3D printing method is developed to fabricate the polymeric stents. Surface modification of polymeric stent is performed by immobilization of 2-N, 6-O-sulfated chitosan (26SCS). Physical and chemical characterization results showed that the surface microstructure of 3D-pinted PCL stents can be influenced by 26SCS modification, but no significant difference was observed for their mechanical behavior. Biocompatibility assessment results indicated that PCL and S-PCL stents possess good compatibility with blood and cells, and 26SCS modification can enhance cell proliferation. These results suggest that 3D printed PCL stent can be a potential candidate for coronary artery disease by modification of sulfated chitosan (CS).

## Introduction

Coronary artery disease is a leading killer of human life in the world, and percutaneous coronary intervention (PCI) with stent implantation has become a standard treating method to restore the blood flow. Bioresorbable stents (BRSs) have attracted great attention as potential candidate in treatment of coronary artery disease, because they are made of bioresorbable materials that can dissolve or be absorbed in the human body, which can provide the mechanical support and then disappear without the occurrence of long-term complications (Waksman, 2006). However, there are still some challenges in BRSs technology, such as manufacturing technique and long-term biocompatibility.

Polycaprolactone (PCL) has become potential material candidate for medical application due to its good ductility, processing property, biodegradability, and biocompatibility. Generally, PCL has a long degradation time (2–3 years) due to the low crystallinity degree (Lakshmi and Cato, 2007). PCL has been widely applied in form of microspheres, nanospheres, and implants. The porous PCL-based scaffolds were widely investigated for bone tissue engineering, and *in vitro* safety and efficacy results showed that they had excellent mechanical properties, biocompatibility, and bioactivity (Mondrinos et al., 2006; Puppi et al., 2012; Khoshroo et al., 2016). Vurugonda et al. (2017) developed copolymer of poly-lactic acid (PLLA) and PCL stents for cardiovascular application. Results highlighted that the mechanical strength, degradation behavior, and drug release behavior of stents were significantly improved, while their samples were fabricated in tabular design which is not suitable for stent application. The braided PCL stents showed superior compression properties and recovery ability by incorporating PPDO monofilaments and annealing, but their degradation rates were attenuated under dynamic loading (Zhao et al., 2018, 2019). Kim et al. (2019) investigated drug-eluting PCL mesh network tubular stent for salivary gland disease. Results showed that their mechanical properties were improved by incorporating antibiotic particles, and a sustained drug release was observed. However, the elongation ratios of drug-eluting stents were dramatically decreased which may limit their expansion behavior.

Extensive research has been carried out to manufacture metallic stents by continuous or pulsed laser cutting, including 316LVM steel, magnesium alloy, and CoCr alloy (Raval et al., 2004; Demir et al., 2012; Demir and Previtali, 2017). Results showed that excellent geometries of stents were obtained, while the desired surface quality required selective process gas, acid pickling, and electrochemical polishing. Flege et al. (2012) first developed polymeric stents by selective laser melting technique. Results suggested that the as manufactured polymeric stents are biocompatible at interaction with human coronary artery smooth muscle cells. Guerra and Ciurana (2017) and Guerra A. J. et al. (2017b) introduced the application of fiber laser cutting on PCL stent manufacture, and addressed that the *in vitro* degradation of stent can be accelerated by increasing input energy density. Although laser cutting is capable to fabricate metallic or polymeric stents, there is still a challenge for manufacturing composite material stents. Moreover, laser cutting is a thermal process, which may cause thermal problems including heat affected zones, micro cracks, and dross deposition.

Recently, the 3D printing technique has been considered as an alternation in stent industry, due to its advantages in design personalization. Park et al. (2015) fabricated a helical drug-coated PCL stent by using 3D rapid prototyping, and good results were obtained from degradation test and animal experiments. 3D printing technique was also used to fabricate PCL-GR, PCL/PLA composite stents (Guerra A. et al., 2017a; Misra et al., 2017). Physical and chemical results showed that 3D printing process had strong effects on the dimensional precision but little influence on the material structure of stents (Guerra and Ciurana, 2018). Biological and mechanical analysis results showed good agreement with rigorous requirements of BRSs (Guerra et al., 2018a). These results also suggested that 3D printing process was highly suitable for manufacturing composite stents. To the best knowledge of author, the application of 3D printing process in stent manufacture is still in the early stage, and further research needs to be developed in terms of machining quality and biocompatibility assessment for BRSs.

Stents come in contact with blood, and incompatibility can cause short-term and long-term complications, such as vessel damage, restenosis, thrombosis, and inflammation (Hu et al., 2015). To overcome these problems, stent modified with chitosan (CS) may be a potential method to improve the bioactivity of cardiovascular remodeling and neointimal formation. CS is a common heparin-like polysaccharide, which has attracted enormous interest for several features, including hydrophilicity, biocompatibility, biodegradability, and bioactivity (Kumar, 2000). 2-*N*, 6-*O*-sulfated CS (26SCS) modified PLGA scaffolds have been widely investigated to improve the bioactivity of vascular endothelial growth factor (VEGF), heparin-binding epidermal growth factor (HB-EGF), bone morphology protein-2 (BMP-2) in angiogenesis, wound healing, and bone regeneration, and the desired results were obtained from morphological observation, releasing profiles, and bioactivity assessment (Kong et al., 2014; Yu et al., 2015, 2018; Peng et al., 2017). 26SCS modified PCL scaffolds were also studied as bone BMP-2 delivery vehicle to accelerate osteoinduction, and results highlighted that 26SCS modified PCL scaffold may enhance the cellular response, release behavior, and osteoinductive activity (Cao et al., 2017). 26SCS and PCL nanofibers were used to fabricate bioactive nanocomposite scaffolds, which can enhance osteoblast-like cells viability and attachment (Ghaee et al., 2017). Overall, blends of 26SCS and other polymers have shown potential improvement in mechanical properties, cell adhesion, and cell proliferation. Therefore, 26SCS modification shows great potential to enhance biocompatibility of cardiovascular stents. However, there has been little research focusing on the fabrication and characterization of 26SCS modified PCL stents.

In this study, 3D printing technique is used to produce PCL stent, and sulfated CS is used to modify the surface of PCL stent. Physical and chemical feature of sulfated CS is characterized by ^13^C NMR test, elemental analysis, Fourier transform infrared spectroscopy (FTIR), and differential scanning calorimetry (DSC). The 3D-printed PCL and S-PCL stents are analyzed by scanning electron microscopy (SEM), mechanical test, degradation test, and biocompatibility assessment to investigate their morphology, mechanical property, blood, and cell compatibility. Moreover, the effects of sulfated CS on stent performance are presented by comparison between PCL and S-PCL stents.

## Materials and Methods

### Materials

Commercially available PCL was purchased from Maya Reagent Co., Ltd. (Zhejiang, China). It is a biodegradable polymer with a glass transition temperature of −60°C and a melting temperature of 60°C. CS (95% deacetylated, Mw 10∼20 × 10^4^ Da) was purchased from Shanghai Macklin Biochemical Co., Ltd. (Shanghai, China). Chlorosulfonic acid (HClSO_3_), N, N-dimethylformamide (DMF), and ethylenediamine (ED) were provided by Shanghai Aladdin Bio-Chem Technology Co., Ltd. (Shanghai, China). Sodium hydroxide (NaOH) and ethanol were provided by Guangzhou Chemical Reagent Factory (Guangzhou, China). Mouse fibroblasts (L929) were obtained from Guangzhou Military Hospital. All cell-culture related reagents were obtained from Gibco (Grand Island, NY, United States).

### Synthesis of Sulfated Chitosan

2-N, 6-O-sulfated chitosan was synthesized according to previous method (Zhou et al., 2009; Cao et al., 2014). In brief, sulfating reagent was prepared by adding 5 mL HClSO_3_ dropwise to 20 mL DMF cooled at 0°C, and the mixture was stirred for stabilization. CS suspension was prepared by mixture with DMF and kept overnight. Sulfating reagent was added in the CS suspension for reaction, kept at 70°C for 4 h. At the end of reaction, the obtained mixture was cooled to room temperature, neutralized with NaOH, and precipitated with ethanol. The 26SCS powder was obtained after the precipitate was dissolved in deionized water, dialyzed, and lyophilized for 2 days.

### Fabrication of PCL Stents

**FIGURE 1:**
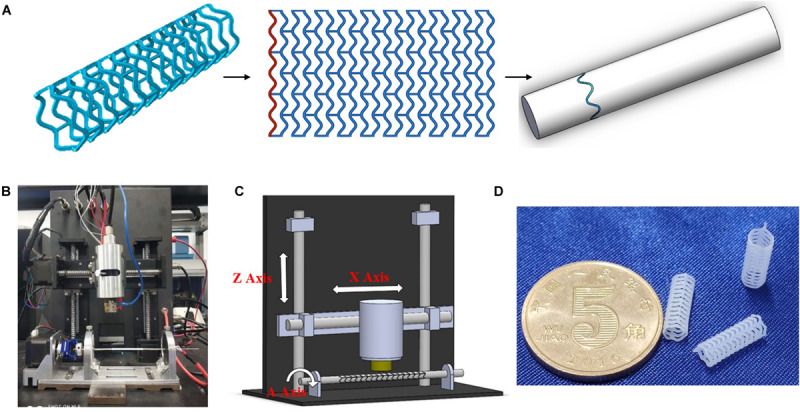
3D-printing trajectory strategy **(A)**, 3D printer machine **(B)**, machine methodology **(C)**, and 3D-printed PCL stents **(D)**.

3D printing method was used to produce a tabular stent with a diameter of 3 mm and a length of 10 mm. As shown in Figure 1, the 3D printing machine is based on the electrospinning. The machine provides a maximum X axis translation of 50 mm, a needle temperature of 200°C, a pressure of 600 kPa, and a repositioning precision of 20 μm. The 3D printing process requires the conversion of PCL particles to filaments. The particles are melted in heating chamber, and then transferred to the extruder needle at pressure of 7 kPa. The produced filaments are deposited onto a controlled metallic rotatory mandrel under voltage of 4 kV.

Screening experiments were performed to optimize the processing parameters for 3D printing of PCL stents. 3D printing trajectory (as shown in Figure 1A) was applied to manufacture the stent uniformly. As a result, the stents were printed at 90°C needle temperature, 1000 mm/min translational velocity, and 1000 r/min rotational velocity (Table 1).

**TABLE 1:** Optimized processing parameters for 3D printing of PCL stents.

**Stent**	**Z(mm)**	**V_T_ (mm/min)**	**V_R_ (r/min)**	**Mandrel diameter**	**Mandrel material**
PCL	1.5	1000	1000	2.5	Brass

*Z: distance between needle and mandrel, V_*T*_: needle translational velocity, V_*R*_: mandrel rotational velocity.*

### Sulfated Chitosan Immobilization on PCL Stents

**FIGURE 2:**
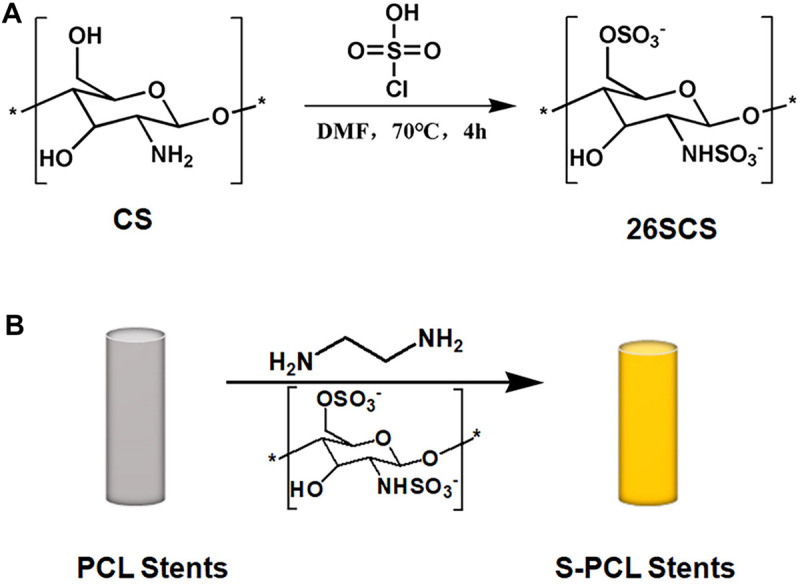
Schematic reaction of 2-N, 6-O-sulfated chitosan **(A)** and aminolysis reaction of sulfated chitosan with PCL stents **(B)**. * means the repetition of molecular structure.

According to the previously described method of Croll et al. (2004), 26SCS immobilization was carried out after the aminolysis of PCL stent (Figure 2). Specifically, PCL stents were soaked in ED with a concentration of 0.2 mol/L for 2 h at room temperature. Stents were washed by ice water for the desired time period, and then immersed in 26SCS solution with a concentration of 10 mg/mL for 4 h. Afterward, the aminated stents were washed repeatedly to remove the redundant 26SCS and dried in a vacuum oven for 24 h at 37 °C. Then the modified stents were stored in a desiccator for further use, and named as S-PCL stents. The unmodified stents were used as negative control, and named as PCL stents.

### Characterization of 2-N, 6-O-Sulfated Chitosan

^13^C NMR spectra were measured by instrument (AV III 500 MHz, Bruker, Germany), in deuterated acid solvent with a concentration of 5%. Elemental analysis (Vario EL Cube, Elementar, Germany) was performed to evaluate the mass proportion changes of carbon (C), nitrogen (N), hydrogen (H), and sulfur (S) in 26SCS. FTIR spectroscopy (Tensor 27, Bruker, Germany) was used to determine the structural changes of CS during the synthesis process. DSC (STA 449 F3, NETZSCH, Germany) was carried out to record the mass change of CS and 26SCS with the increase of temperature.

### Characterization of PCL and S-PCL Stents

#### Morphological Observation of Stents

The 3D-printed PCL and S-PCL stents were morphologically characterized by SEM (S3400, Hitachi, Japan). The stents were sputter coated with gold and mounted on the instrument plate by conductive coating, and then the surface morphology was captured and analyzed with different magnifications at an accelerating voltage of 5 kV.

#### Mechanical Characterization of Stents

The mechanical properties were evaluated by lateral crush resistance tests on both PCL and S-PCL stents at room temperature. Five parallel tests were conducted for each group of stents on mechanical test instrument (ElectroForce 3220, Bose, United States). The stent was placed between two plates of testing machine, and compressed by controlling the constant displacement of the upper plate at a rate of 1 mm/min to a displacement of 2 mm. The load–displacement curve was measured directly from the instrument, and then the crush resistance stiffness was extracted.

#### *In vitro* Degradation Assessment

The dried S-PCL stents were incubated with phosphate buffered saline (PBS, ST476, Beyotime, China) at 37°C, after the initial weight was recorded. The volume ratio of PBS and sample is required to be at least 30:1, to make sure that samples are totally immersed in PBS solution. Lysozyme was added to accelerate the degradation of S-PCL stents. At predefined time intervals (*t* = 10, 20, 30, 40, 50, 60 days), samples were weighed to record their final weights. The degradation rate is calculated by the following equation:

(1) $$Degradation\left(\%\right)=\frac{m_{i}-m_{f}}{m_{i}}\times 100\%$$

Where the *m*_i_ and *m*_f_ represent the initial weight and final weight, respectively.

### Blood Compatibility Assessment

The extract tests were used to evaluate the biocompatibility of PCL and S-PCL stents, according to ISO 10993-12 (2012). The PCL and S-PCL stents were immersed in serum-free Dulbecco’s modified Eagle’s Medium (DMEM) and placed in a container at 37°C for 24 h to obtain the extracts of stents. Different concentrations of extracts were obtained by diluting the mother liquor with DMEM, and then stored at 4°C for further use.

#### *In vitro* Hemolysis Assay

In this study, fresh whole blood of male Sprague–Dawley rats was used to assess the blood compatibility of PCL and S-PCL stents. In this assay, 4 mL extracts of PCL and S-PCL stents were incubated with 200 μL suspension of 16% red blood cells (RBCs) suspension in centrifuge tubes, respectively. While the deionized water and PBS were used as positive and negative controls, respectively. Three parallel tests were carried out for each group. Then the test samples were centrifuged under 1000 *g* for 5 min to collect the supernatant at predefined time points of 1, 3, 5, 8, 18, and 24 h. The optical density (OD) of RBCs was measured at 540 nm by microplate reader (MULTISKAN MK3, ThermoFisher).

#### Morphology of RBCs

The whole blood was centrifuged under 1000 *g* for 5 min, and then the RBCs were collected. The extracts of PCL and S-PCL stent (1 mL) were mixed and incubated with RBCs (50 μL) for 1 h at 37°C, centrifuge the mixture, and remove the supernatant to obtain RBCs. After washing RBCs with PBS, 4% paraformaldehyde was added to fix cells for 1 h. Subsequently, the fixed RBCs were dehydrated using ethanol of 70, 85, 95, and 100% in turn. Finally, the SEM was conducted to observe the morphology of RBCs.

#### Blood Coagulation Assay

The whole blood was centrifuged under 1000 *g* for 5 min, and the upper serum was collected for testing. The extracts of PCL and S-PCL stent (30 μL) were mixed and incubated with serum (270 μL) for 10 min at 37°C. The activated partial thromboplastin time (APTT) and prothrombin time (PT) were measured by automatic hematology analyzer (BC-5000, Mindray, Shenzhen). It should be noted that PBS was used as negative control in this assay.

### *In vitro* Biocompatibility Assessment

#### Cell Viability

Mouse fibroblasts (L929 cells) were cultured in 96-well plate in DMEM at a density of 5×10^3^ cells/well, and allowed to attach overnight at 37°C in a humidified environment of 5% CO_2_, and the medium of DMEM was supported with 10% fetal bovine serum and 1% penicillin/streptomycin. Subsequently, DMEM was removed and replaced with series of extract solution. Negative control was designed by cell culture with DMEM and PBS. Pure DMEM and PBS were used as positive control and negative controls, respectively. After 24 h of culturing cells, L929 was washed using PBS, and fresh DMEM (containing 10% CCK-8) was added into each well. After 1 h incubation, OD of each cell was measured at 450 nm using a microplate reader.

#### Live/Dead Staining

The 3D-printed PCL and S-PCL stents were immersed in 75% ethanol, sterile PBS, and DMEM medium in turn for 1 h to sterilize, respectively. L929 cells at a concentration of 2 × 10^5^/mL were seeded on the surface of sterilized stents and cultured at 37°C in a humidified environment of 5% CO_2_ for 7 days. The Calcein-AM and propidium iodide (PI) were added for staining, and the L929 cells Live/Dead staining images were obtained by inverted fluorescence microscope (TE2000-S, Nikon, Japan).

#### Cytoskeletal Immunofluorescence Staining

Phosphate buffered saline solution was used to wash the L929 cell-loaded stents for three times, and paraformaldehyde with a concentration of 4% was added to fix for 0.5 h. The stents were washed with PBS for another three times, and then treated with 0.5% Triton-X-100, phalloidin, and DAPI for 10, 30, 10 min, respectively. Finally, anti-fluorescence quencher was added and stored in dark at 4°C. The immunofluorescence staining images were captured by laser confocal microscope (FV3000, Olympus, Japan).

#### Cell Proliferation

The L929 cells with a concentration of 2 × 10^4^/mL were seeded on the surface of sterilized stents and incubated at 37°C in a humidified environment of 5% CO_2_. The CCK-8 reagent was added to each stent, and the OD of stent at 1, 3, and 7 days was measured at 450 nm using a microplate reader.

## Results and Discussion

### Characterization of Sulfated Chitosan

Supplementary Figure S1 is the result of the ^13^C NMR spectra of 26SCS. The magnitudes of chemical shift in 26SCS are in good agreement with the data in the previous report (Vikhoreva et al., 2005). There are two peaks at 69 and 60 ppm which represents C6 and C2 of 26SCS, respectively. The stable signal at C2 confirms the low sulfation of aminogroups in synthesized sulfated CS. This suggests that the sulfonation reaction occurs at the location of C6 and C2 of 26SCS.

The mass proportion is 33.19, 5.49, 2.63, and 5.52% for carbon (C), nitrogen (N), hydrogen (H), and sulfur (S), respectively, as shown in Supplementary Table S1. The appearance of sulfur (S) confirms the successful synthesis of 26SCS by using HClSO_3_. The calculated degree of sulfonation is 44%.

The FTIR spectra of CS and 26SCS powders are shown in Supplementary Figure S2. There are two significant absorption peaks at 1220 and 800 cm^–1^ for 26SCS compared with CS. The absorption peak at 1220 cm^–1^ can be attributed to stretching vibrations of O = S = O groups, and the peak at 800 cm^–1^ are related to stretching vibrations of C-O-S groups (Ghaee et al., 2016). The occurrence of new peaks indicated that synthesis of sulfated CS was successful.

Supplementary Figure S3 shows the mass change of CS and 26SCS with the increase of temperature. There are two stages during the mass loss process of CS and 26SCS. During the first stage, mass loss is mainly caused by water loss, where CS and26 SCS possesses 4 and 9% drop for mass, respectively. Afterward, their mass loss has a dramatic drop to 50%, which results in degradation. It can be found that 26SCS has a faster degradation rate than that of CS, which might be caused by the degradation of sulfonic acid groups.

### Characterization of Stents

#### Morphology Observation of Stents

**FIGURE 3:**
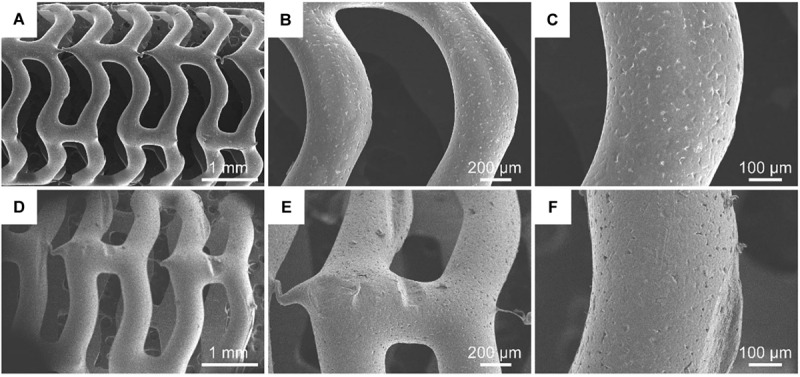
SEM images of PCL stent **(A–C)** and S-PCL stent **(D–F)** with different magnifications.

Figure 3 shows the surface morphology of PCL and S-PCL stents with different magnifications. The 3D-printed PCL and S-PCL stents behaved in tabular shapes with uniform diameters. Under large magnification, the surface of PCL stent was smooth, but the S-PCL stent had a rough and porous surface. This result indicates that the stent surface quality is affected by modification of 26SCS.

The surface quality of stent over the manufacturing process makes effect on their blood compatibility, anti-thrombosis, and vessel healing after deployment in human body. Guerra et al. (2018b) reported that ultraviolet sterilization method can increase surface roughness of 3D-printed PCL stent, and further influence its mechanical properties and degradation behavior. It was also reported that rough surface of stents can highly improve endothelial cell attachment and growth, and smooth surface contributes to endothelia cell migration (Lutter et al., 2014). These results highlight the importance of surface morphology characterization for medical devices. It should be noted that the 26SCS modification can produce a relative rough surface, and this may improve the biological performance of PCL stents.

#### Mechanical Properties of Stents

**FIGURE 4:**
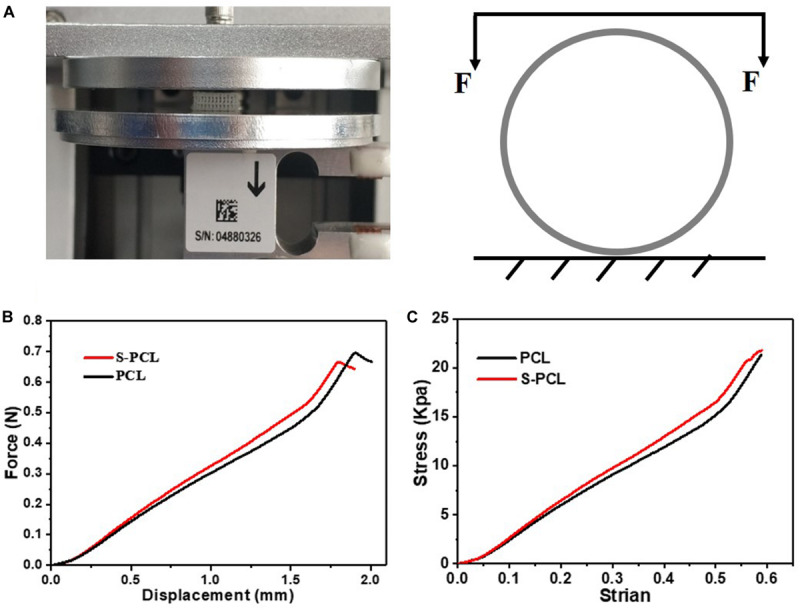
Lateral crush resistance test **(A)**, force–displacement curve **(B)**, and stress–strain curve **(C)**.

Figure 4A shows the testing machine and schematic diagram for lateral crush resistance test, and Figures 4B,C show the force–displacement behavior and stress–strain behavior for PCL and S-PCL stents, respectively. There was no significant difference on the force–displacement or stress–strain behavior between PCL and S-PCL stents, which suggested that 26SCS modification made no effect on the mechanical properties of PCL stents.

Bioresorbable stents undergo large deformation and high stresses while being implanted in diseased vessels under cyclic pulsatile loadings, and therefore the assessment of mechanical properties is crucial in stenting technology. In this study, lateral crush resistance tests were performed to simulate the *in vivo* loadings experienced by stents. The obtained mechanical results were similar with those reported for poly (L-lactic-acid) stents by Wang et al. (2018). It is a fact that bioresorbable polymer has low mechanical strength compared to metal. However, the mechanical properties of these polymers are more dependent on their microscopic characteristics, such as degree of crystallinity, molecular weight, and chemical structure, which make it easy for material processing and improvements. Therefore, bioresorbable polymers such as PCL and PLLA have been considered as potential material candidates for stents.

#### *In vitro* Degradation Study

**FIGURE 5:**
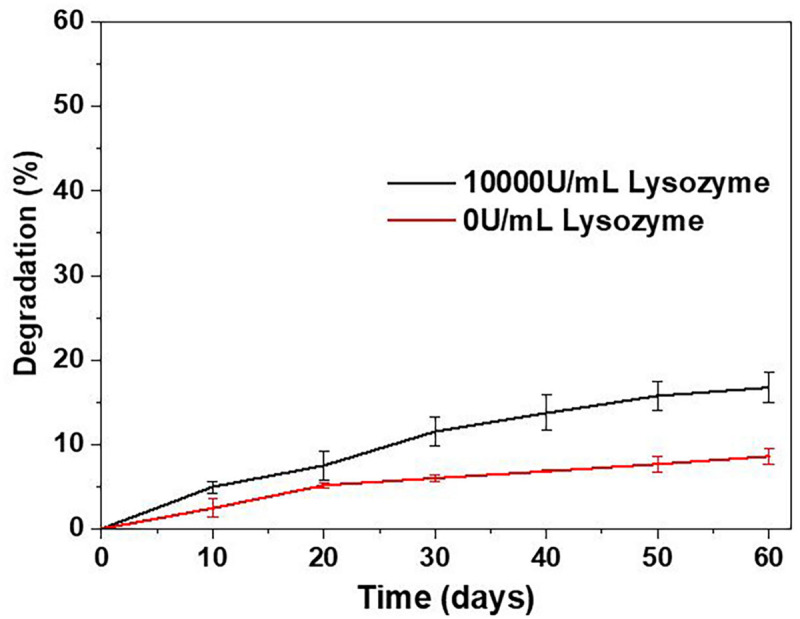
Degradation behavior of S-PCL stents.

The mechanical properties of BRSs are highly affected by the degradation process. Figure 5 shows the *in vitro* degradation behavior of S-PCL stents with and without lysozyme. The S-PCL stent showed gradual mass decrease with the increase of degradation time. The weight loss of S-PCL is 16 and 7% with and without lysozyme at 60 days, respectively. It is obvious that lysozyme can accelerate the degradation of S-PCL stent at body temperature.

It is well known that degradation of polymer occurs by the way of hydrolysis and enzymatic degradation. Generally, PCL mainly undergoes hydrolytic degradation by the breakup of ester groups, and then experience intracellular degradation when it is highly crystalline with low molecular weight (Woodruff and Hutmacher, 2010). It is reported that the degradation rate of PCL is extremely low, and the total *in vivo* degradation takes at least 2 years (Sun et al., 2006; Zong et al., 2015). The addition of lysozyme sped up degradation rate by two times, as show in our degradation result. It can be concluded that enzymatic degradation is faster than hydrolytic degradation for PCL. Therefore, it might be useful to modify the degradation rate of polymer by adding suitable aseptic and non-toxic enzyme, such as lysozyme.

### Blood Compatibility Assessment

#### *In vitro* Hemolysis Analysis

**FIGURE 6:**
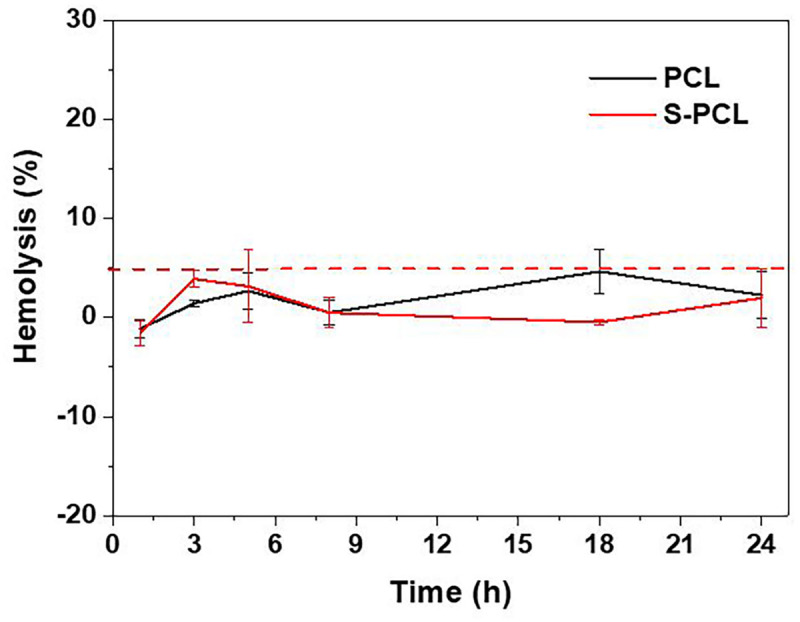
Hemolytic percentage of RBCs incubated with PCL and S-PCL stent extracts at different time points.

The polymeric stents are deployed in human blood vessels for the treatment of coronary artery disease, and therefore the blood compatibility assessment is essential. RBCs, as one of key components in whole blood, usually have a volume fraction of 40–50%, and its principal function is to deliver oxygen to the tissues by blood flow through circulatory system. Hemolysis test can produce deep understanding of complicated interactions between foreign material and RBCs membrane, and thus often used to assess the hemocompatibility of biomedical polymers. In this study, the *in vitro* hemolysis assay was conducted to investigate the influence of PCL and S-PCL stent extracts on the hemolytic behavior of RBCs. Generally, material is considered as non-hemolytic with hemolysis below 2%, and hemolytic but acceptable whereas the hemolysis between 2 and 5% (Vurugonda et al., 2017). The result showed that all test samples produced hemolysis below 5%, which indicated that both PCL and S-PCL stents possess good hemocompatibility, as shown in Figure 6.

#### Morphology of RBCs

**FIGURE 7:**
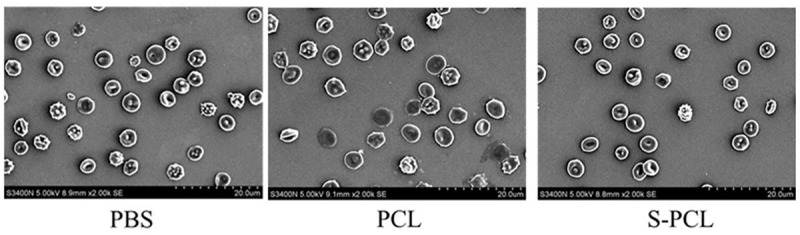
Morphology of RBCs in extracts of PCL and S-PCL stent and PBS solution.

Scanning electron microscopy was used to investigate the influence of PCL and S-PCL stent on the morphology of RBCs, and PBS was used as control. Normally, RBCs behave in circular shape with concaves, which makes easy to identify the morphological change by interaction with foreign materials. As shown in Figure 7, there is no aggregation of RBCs, and the morphology is as normal as that of PBS control. The result reveals that the PCL and S-PCL stents have little impact on the morphology of RBCs.

Liu et al. (2012) reported that interactions between foreign materials and RBCs are caused by hydrophobic function with the lipid bilayer and electrostatic function with surface charges. With the increase of hydrophobicity, biomaterials may partition into the RBCs membrane and destroy the lipid bilayer, which lead to hemolysis of RBCs. In this study, PCL is considered as hydrophobic polymer, but there is no morphological change observed while interacting with RBCs. As reported, the effect of hydrophobized polymer on RBC morphology is dependent on the concentration of polymers (Liu et al., 2010). Therefore, a relatively low concentration of PCL and S-PCL stent extracts may hardly result in the morphological change of RBCs.

#### APTT/PT

**FIGURE 8:**
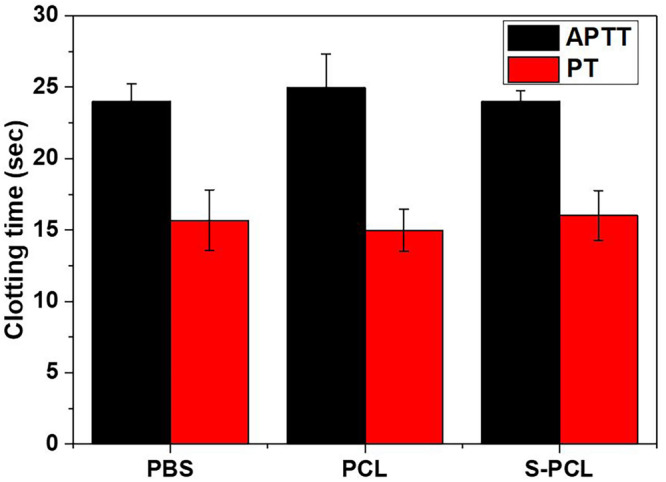
Effect of PCL and S-PCL stent on APTT and PT compared to PBS control.

The APTT and PT are generally used to evaluate the blood coagulation function. The results showed that there are no significant differences on APTT and PT magnitudes between PCL and S-PCL stents with PBS control (Figure 8), which suggested that PCL and S-PCL stents have good blood compatibility.

Generally, bioresorbable polymers are considered as non-toxic and less likely to cause severe inflammatory response. However, the cytotoxic effect of biodegradable polymer on cells was proved to be dependent on their components, molecular weight, and manufacturing technique. Wang et al. (2014) reported that PLLA/PCL microparticles with low molecular weight induced cytotoxicity and prevented endothelial cell function, while Kang et al. (2008) found that high molecular weight PLLA and PLGA particles were non-toxic to cancer cells. Stents, made by blending PLLA and PCL, were proved to minimize the local acidification and the chronic inflammatory response (Liao et al., 2008). Nanofibrous PLACL/collagen stents possess admirable cardiac cell attachment and growth, compared to direct synthetic PLACL blends (Mukherjee et al., 2011). Therefore, biocompatibility tests were carried out to investigate the biocompatibility of 3D-printed PCL and S-PCL stents, in terms of cell viability assay, hemolysis assay, and blood coagulation assay. All these results showed that both PCL and S-PCL stents are non-toxic and blood compatible, which suggest that 3D-printed stents can be potential biomedical devices in coronary vessels.

### *In vitro* Biocompatibility Assessment

#### *In vitro* Cytotoxicity Analysis

**FIGURE 9:**
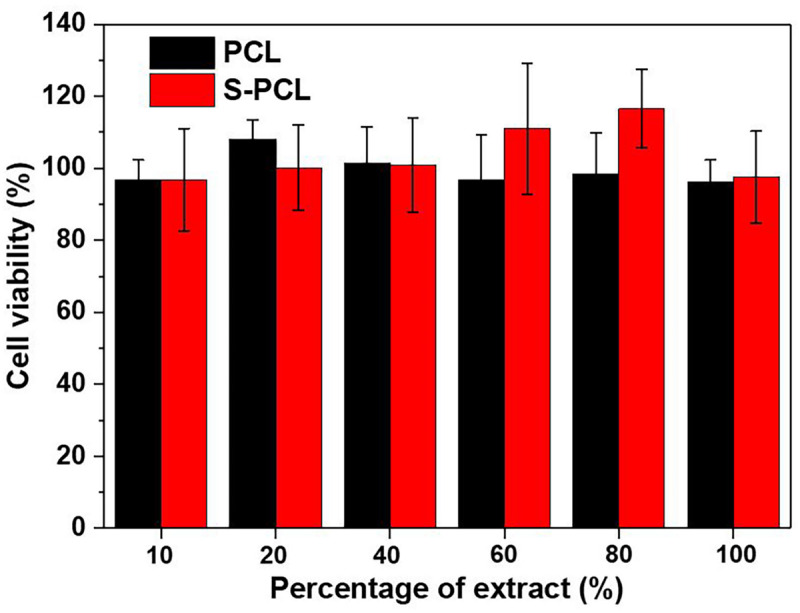
Cell viability with different percentages of PCL and S-PCL stent extracts.

The *in vitro* cytotoxicity is an important biological parameter in the evaluation of biocompatibility of medical devices. Standard CCK-8 assay was carried out to evaluate the L919 cell viability in incubation with the extracts of PCL and S-PCL stents, as shown in Figure 9. The cytotoxic assay indicated that all 3D-printed PCL and S-PCL stents were non-toxic to L929 cells. There was no significant difference of the cell viability between PCL and S-PCL stents, which suggested that both PCL and S-PCL stents were compatible with cell culture. It should be noted that the cell viability of S-PCL stents was higher than that of PCL stents at 60%, 80% concentration of extract, with an approximately 20% increase, which indicated that cell proliferation was enhanced. This positive effect may be caused by 26SCS modification, and thus 26SCS might contribute to enhancement of cell proliferation.

#### *In vitro* Live/Dead Staining

**FIGURE 10:**
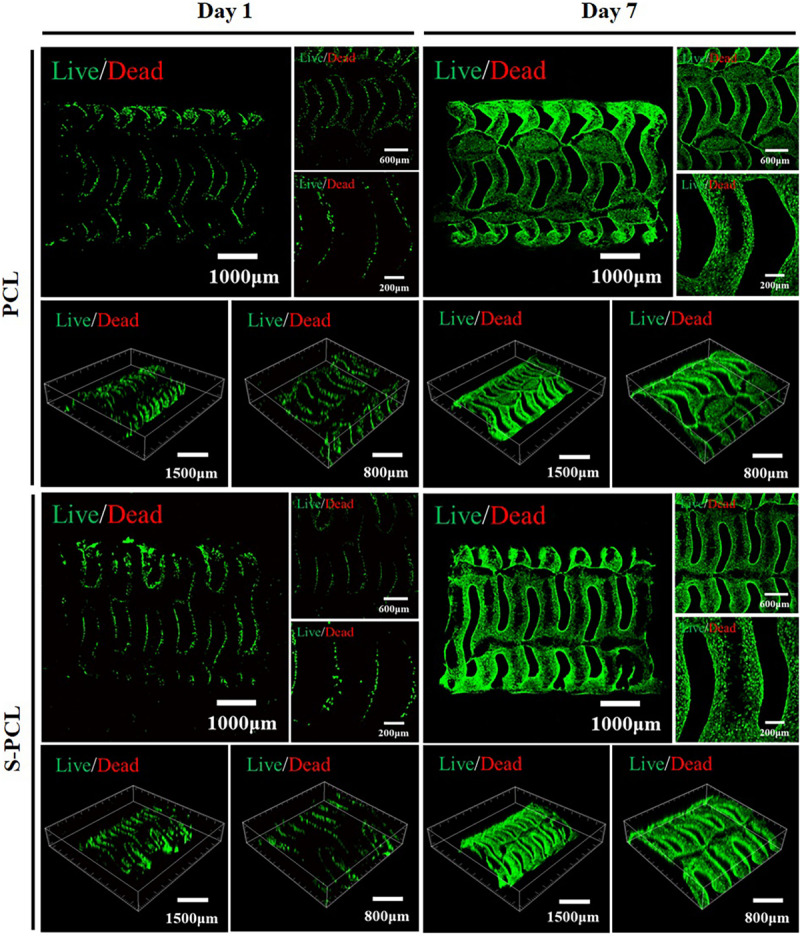
Live/Dead staining images of L929 cells seeded on the PCL and S-PCL stents after 1 and 7 days.

Figure 10 shows the Live/Dead staining images of PCL and S-PCL stents after cell culture of 1 and 7 days. The live cells were stained to green and the dead cells were stained to red by Calcein-AM and PI, respectively. It is clear that L929 cells show increasing survival rates on both PCL and S-PCL stents when the cell culture time increases from 1 to 7 days. Moreover, the cells-loaded S-PCL stents obtained a higher survival rate than PCL stents at 7 days. This indicates that both 3D-printed PCL and S-PCL stents have excellent cells compatibility, and S-PCL stents show potential enhancement of cell adhesion and growth.

#### Cytoskeletal Immunofluorescence Staining

**FIGURE 11:**
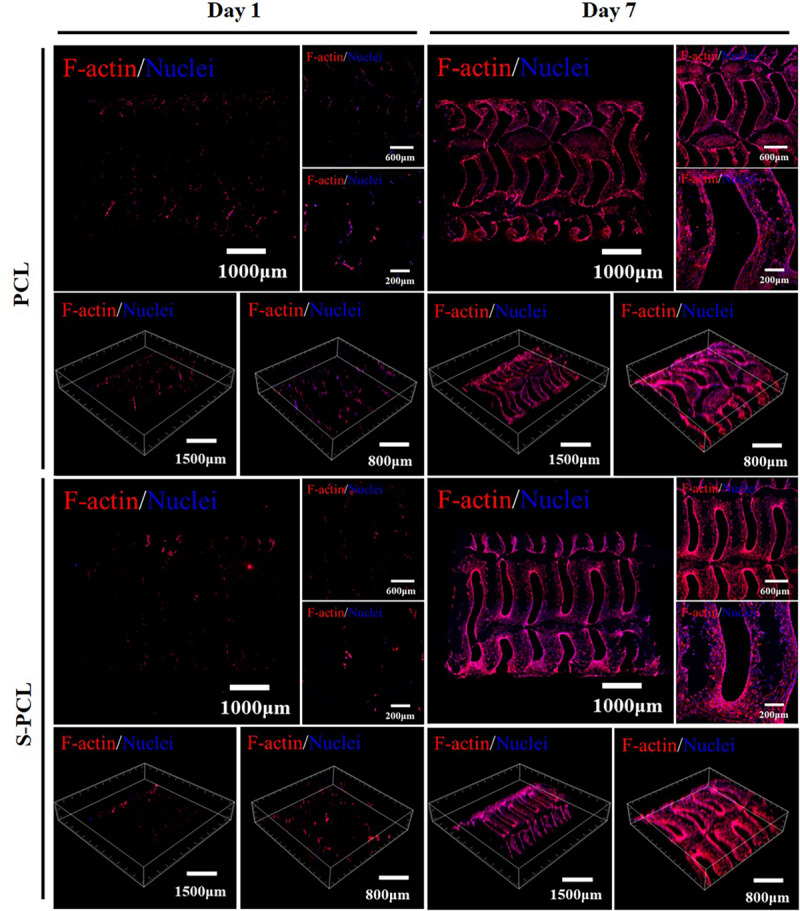
Cytoskeleton fluorescence staining images of L929 cells seeded on PCL and S-PCL stents after 1 and 7 days.

Figure 11 shows the cell adhesion and coverage on the PCL and S-PCL stents after cell culture of 1 and 7 days. The red fluorescent labeled F-actin grows in more and more uniform distribution with the increase of cell culture time, which suggests that the cells behave in extended state and firmly attach to the surface of PCL and S-PCL stents. The coverage of L929 cells on S-PCL stents is also slightly higher than that of PCL stents at 7 days. This suggests that the surface of 3D-printed PCL and S-PCL stents is favorable for cell proliferation.

#### *In vitro* Cell Proliferation

**FIGURE 12:**
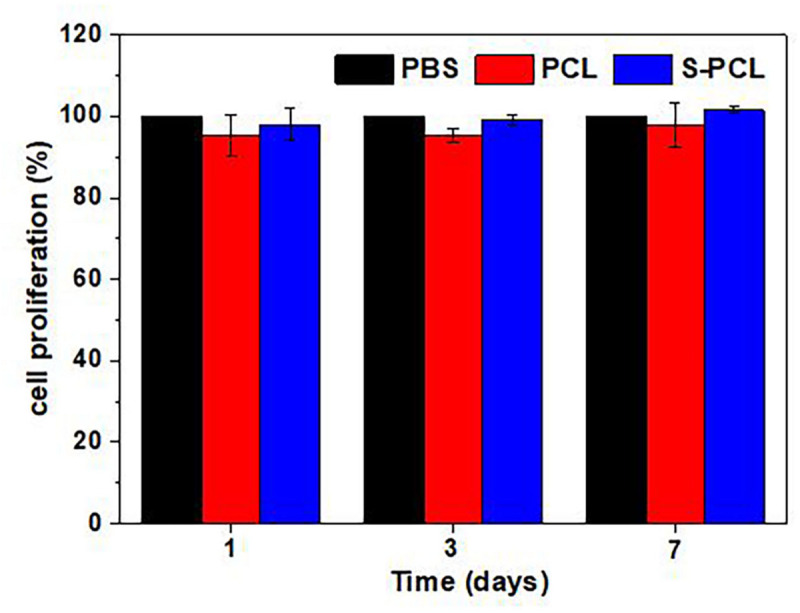
Cell proliferation on PCL and S-PCL stents compared to PBS control.

The standard CCK-8 assay was also used to evaluate the cell proliferation on PCL and S-PCL stents, and results are shown in Figure 12 at 1, 3, and 7 days. It was found that the cells seeded on both PCL and S-PCL stents produced high proliferation rate over the culture period. There was no significant difference between two types of stents, compared to the control group. This suggested that both PCL and 26SCS were favorable for the cell attachment and growth.

## Conclusion

In this study, a novel PCL stent was fabricated by 3D printing technique. 26SCS was successfully synthesized and used to modify the surface of PCL stent. Physical characterization results showed that PCL stent obtained good surface which is suitable for endothelial cell attachment and growth, by surface modification of 26SCS. The mechanical property is comparable to that of the existing bioresorbable polymeric stents. The degradation rate of PCL stent was improved by function of lysozyme. *In vitro* data demonstrated that PCL stents possessed non-cytotoxicity, admirable blood compatibility, and excellent cell compatibility, and 26SCS modification could enhance their cell viability and cell proliferation. Altogether, this current study produces that PCL and S-PCL stents can be potential medical devices for coronary artery disease.

## Data Availability Statement

The raw data supporting the conclusions of this article will be made available by the authors, without undue reservation, to any qualified researcher. Requests to access the datasets should be directed to qiutianyangustu@126.com.

## Author Contributions

TQ designed and carried out the experiments and wrote the manuscript. WJ collected the data and drew the figures. PY and LJ developed analysis tools. XW analyzed the experimental results.

## Conflict of Interest

The authors declare that the research was conducted in the absence of any commercial or financial relationships that could be construed as a potential conflict of interest.
