# Supplementary material for: Development of 3D-Printed Sulfated Chitosan Modified Bioresorbable Stents for Coronary Artery Disease
Source: Front Bioeng Biotechnol. 2020 May 19;8:462. doi: 10.3389/fbioe.2020.00462 (PMC7248363; doi:10.3389/fbioe.2020.00462)
Supplement: Supplementary file 1 [file Table_1.DOCX]

Table S1. Elemental analysis and degree of sulfonation of SCS.

| Sample | N (%) | C (%) | S (%) | H (%) | Degree of sulfonation (%) |
| --- | --- | --- | --- | --- | --- |
| SCS | 5.49 | 33.19 | 5.52 | 2.63 | 44 |
