# Supplementary material for: Development of 3D-Printed Sulfated Chitosan Modified Bioresorbable Stents for Coronary Artery Disease
Source: Front Bioeng Biotechnol. 2020 May 19;8:462. doi: 10.3389/fbioe.2020.00462 (PMC7248363; doi:10.3389/fbioe.2020.00462)
Supplement: Supplementary file 2 [file Data_Sheet_1.PDF]

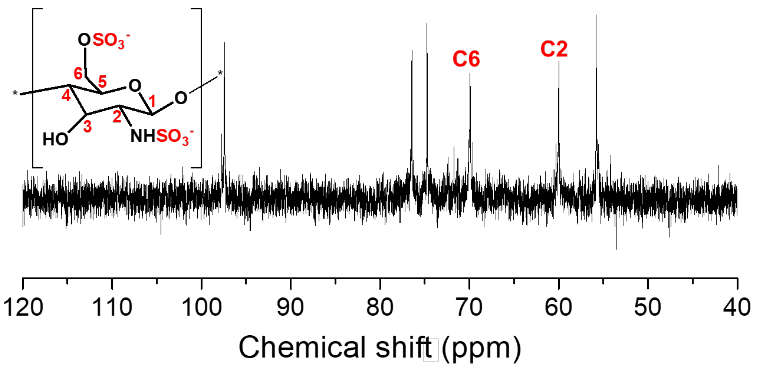

Figure S1. ^{13}C NMR spectra of SCS.

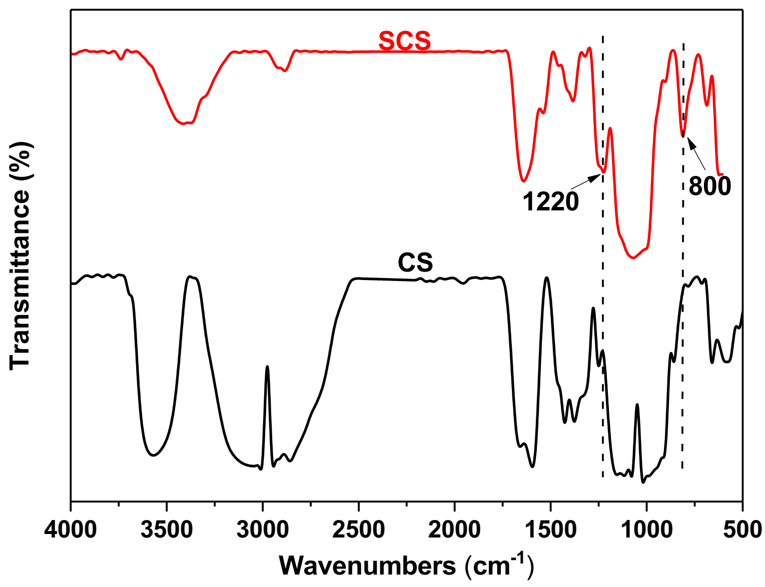

Figure S2. FTIR spectra of CS and SCS.

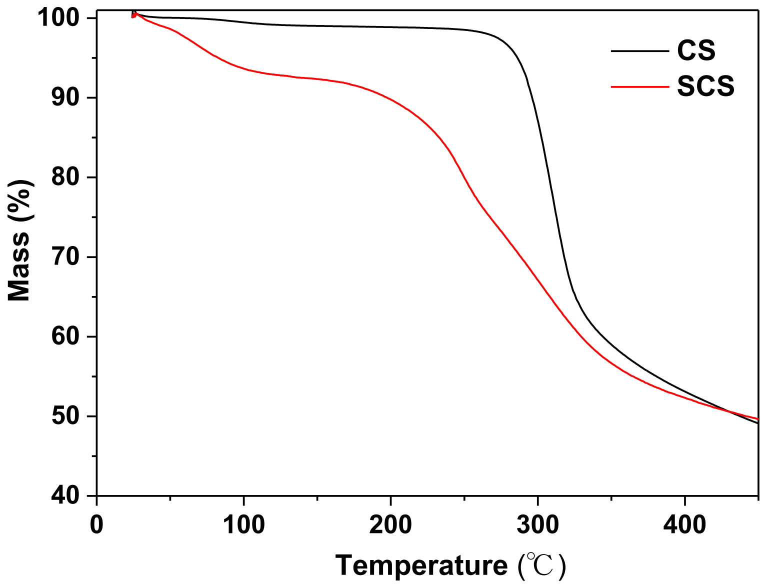

Figure S3. DSC curve of CS and SCS.
